# Supplementary material for: Identification of photoperiod-induced specific miRNAs in the adrenal glands of Sunite sheep (Ovis aries)
Source: Front Vet Sci. 2022 Jul 22;9:888207. doi: 10.3389/fvets.2022.888207 (PMC9354845; doi:10.3389/fvets.2022.888207)
Supplement: Supplementary file 1 [file Data_Sheet_1.ZIP › Supplementary file/miRDeep2 file.zip!/miRDeep2 file/Know_Structure/SPLP42A1/oar-mir-23a.pdf]

miRBase precursor : oar-mir-23a
Total read count : 40387
oar-miR-23a read count : 20212
oar-miR-23b read count : 20170
remaining reads : 5

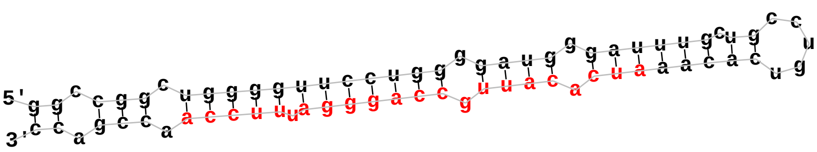

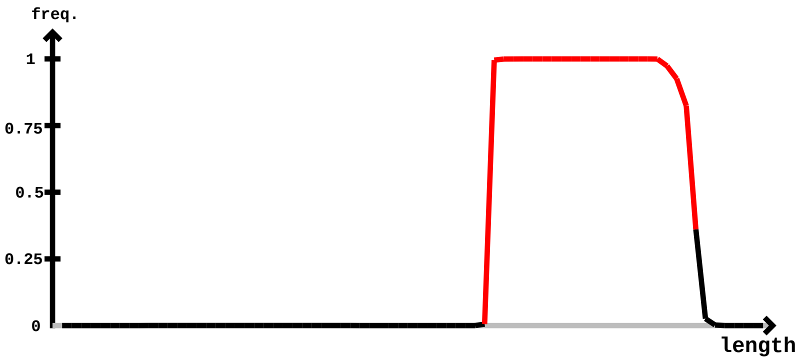

oar-miR-23a
oar-miR-23b

```
5'- ggccggcugggguuccuggggaugggauuugcugccugucacaaaucacauugccagggauuuccaaccgacc   -3'    exp
    ((.(((.((((((((((((.((((.((((((.((.....)))))))).)))).))))))).))))).))).))   reads     mm        sample
    .......ugggguuccuggggaugggauu............................................   1         0         seq
    ........gggguuccuggggaugggauuu...........................................   4         0         seq
    ...........................................aaucacauugccagggauu...........   2         0         seq
    ...........................................aaucacauugccagggauuu..........   10        0         seq
    ...........................................aaucacauugccagggauuuc.........   13        0         seq
    ...........................................aaucacauugccagggauuucc........   65        0         seq
    ...........................................aaucacauugccagggauuucca.......   18        0         seq
    ...........................................aaucacauugccagggauuuccaa......   1         0         seq
    ............................................aucacauugccagggau............   7         0         seq
    ............................................aucacauugccagggaC............   1         1         seq
    ............................................aucacauugccUgggauu...........   2         1         seq
    ............................................aucacauugcUagggauu...........   2         1         seq
    ............................................aucacauugccagggauu...........   516       0         seq
    ............................................aucacauugccagggauuu..........   952       0         seq
    ............................................aucacauugccagggauuuc.........   2030      0         seq
    ............................................aucacauugccagggauuucc........   9283      0         seq
    ............................................aucacauugccagggauuucca.......   6713      0         seq
    ............................................aucacauugccagggauuuccaa......   474       0         seq
    ............................................aucacauugccagggauuuccaac.....   35        0         seq
    ............................................aucacauugccagggauuuccaacc....   1         0         seq
    .............................................ucacCuugccagggauu...........   2         1         seq
    .............................................ucacauugccagggauuu..........   4         0         seq
    .............................................ucacCuugccagggauuu..........   1         1         seq
    .............................................ucacauugccagggauuuc.........   4         0         seq
    .............................................ucacauugccagggauuucc........   29        0         seq
    .............................................ucacauugccagggauuucca.......   27        0         seq
    .............................................ucacauugccagggauuuccaa......   7         0         seq
    .............................................ucacauugccagggauuuccaac.....   6         0         seq
    ..............................................cacauugccagggauuucc........   1         0         seq
    ..............................................cacauugccagggauuucca.......   2         0         seq
    ...............................................acauugccagggauuucc........   2         0         seq
    ...............................................acauugccagggauuucca.......   2         0         seq
```
